# The Impact of Winter Relocation and Depuration on Norovirus Concentrations in Pacific Oysters Harvested from a Commercial Production Site

**DOI:** 10.1007/s12560-018-9345-5

**Published:** 2018-05-03

**Authors:** Agnieszka Rupnik, Sinéad Keaveney, Leon Devilly, Francis Butler, William Doré

**Affiliations:** 1 Marine Institute, Rinville, Oranmore, Ireland; 2 Centre for Food Safety, University College Dublin, Dublin, Ireland

**Keywords:** Human norovirus, RT-qPCR, Oysters, Depuration, Monitoring

## Abstract

Oysters contaminated with norovirus present a significant public health risk when consumed raw. In this study, norovirus genome copy concentrations were determined in Pacific oysters (*Magallana gigas*) harvested from a sewage-impacted production site and then subjected to site-specific management procedures. These procedures consisted of relocation of oysters to an alternative production area during the norovirus high-risk winter periods (November to March) followed by an extended depuration (self-purification) under controlled temperature conditions. Significant differences in norovirus RNA concentrations were demonstrated at each point in the management process. Thirty-one percent of oyster samples from the main harvest area (Site 1) contained norovirus concentrations > 500 genome copies/g and 29% contained norovirus concentrations < 100 genome copies/g. By contrast, no oyster sample from the alternative harvest area (Site 2) or following depuration contained norovirus concentrations > 500 genome copies/g. In addition, 60 and 88% of oysters samples contained norovirus concentrations < 100 genome copies/g in oysters sampled from Site 2 and following depuration, respectively. These data demonstrate that site-specific management processes, supported by norovirus monitoring, can be an effective strategy to reduce, but not eliminate, consumer exposure to norovirus genome copies.

## Introduction

Norovirus infections are the most common cause of non-bacterial gastroenteritis worldwide. In Europe, the peak of illness cases occurs in the winter months, with most patients experiencing relatively mild symptoms. During these epidemic winter periods, the norovirus distribution in the human population, and therefore in sewage, is ubiquitous (Flannery et al. 2012; Kitajima et al. 2012). Filter-feeding bivalve molluscan shellfish such as mussels, clams and oysters can become contaminated with human norovirus when grown in areas impacted by sewage discharges. Such shellfish present an identified public health risk when consumed raw or lightly cooked (Bellou et al. 2013).

The public health risks associated with bivalve molluscan shellfish are clearly recognised and regulations exist throughout the world to manage their production. In Europe, regulatory controls primarily centre around the sanitary classification of harvesting areas into three categories based on *E. coli* concentrations (Anonymous 2004). Each classification category requires harvested shellfish to be treated to differing degrees depending on the level of harvest area contamination. In addition, market-ready oysters must comply with an *E. coli* standard of < 230 MPN/100 g shellfish flesh. Acceptable post-harvest treatments available to ensure oysters meet the *E. coli* standard include self-purification in land-based tanks of clean seawater by a process called depuration, relaying bivalve shellfish in clean seawater areas for an extended period and heat treatment by approved processes. Despite controls, virtually eliminating outbreaks of bacterial illness following shellfish consumption, numerous outbreaks of norovirus gastroenteritis associated with molluscan shellfish consumption continue to occur. In particular, such outbreaks have been associated with the consumption of oysters (Ang 1998; Baker et al. 2010; Chalmers and McMillan 1995; Le Guyader et al. 2008). This is principally because (1) oysters are most often consumed raw, (2) norovirus has been demonstrated to specifically bind to oyster tissues and (3) oysters are grown in intertidal areas often impacted by sewage discharges (McLeod et al. 2017). Norovirus-related gastroenteritis outbreaks have occurred even when oysters have been demonstrated to be fully compliant with regulatory end-product standards (Baker et al. 2010; Chalmers and McMillan 1995; Doré et al. 2010). Therefore, the combination of harvest area controls and post-harvest treatments as currently practiced are not considered to fully protect oyster consumers from the risk associated with norovirus contamination (EFSA 2012).

Controls based on acceptable limits for norovirus are necessary to further protect consumers. Although recognised as being required (EFSA 2012), controls based on virus standards in bivalve molluscan shellfish have not been forthcoming. To some extent, this has been due to the lack of standardised and reliable procedures for the detection and quantification of norovirus in oysters. Such a tool has become available more recently with an introduction of a standardised quantitative real-time reverse transcription PCR (RT-qPCR) method able to monitor norovirus genome copy concentrations (“ISO 15216-1:2017” 2017). It has already been used by us and other laboratories to establish the prevalence and concentration of norovirus in oyster harvest areas (Lowther et al. 2012a) and in outbreak investigations (Baker et al. 2010; Doré et al. 2010).

In this study, we monitored norovirus RNA concentrations in oysters at a production site known to be impacted by sewage, contaminated with norovirus, and previously associated with a large-scale illness outbreak (Doré et al. 2010). Risk management procedures were introduced by the producer during commercial production in response to this outbreak. A two-stage management approach was followed at the site. First, during the high-risk winter period, oysters were only harvested from an alternative local site which had previously been identified as being subject to less norovirus contamination than the main site. Second, following harvest, oysters were subjected to depuration for an extended period of up to 9 days under controlled temperature conditions. During a 14-month period, we used the standardised RT-qPCR procedure to determine norovirus genome copy concentrations in oysters in the main production site, the alternative harvest area, and following depuration to determine the impact of the management procedures on potential consumer exposure to norovirus genome copies.

## Materials and Methods

### Sampling Sites and Management Controls Procedures During Commercial Production

The main harvest site (Site 1) is located in an estuary that has a number of potential sources of sewage contamination. The most significant of them is a secondary wastewater treatment plant (WWTP), serving a population equivalent of 1200 with the discharge outlet located approximately 1.5 km away from Site 1. Further significant contamination sources (such as septic tanks or other wastewater treatment plants) may also impact on the production site but these are more distant. Throughout the study period, this production area was classified as a category A site, based on *E. coli* monitoring under European regulations (Anonymous 2004).

**Fig. 1:**
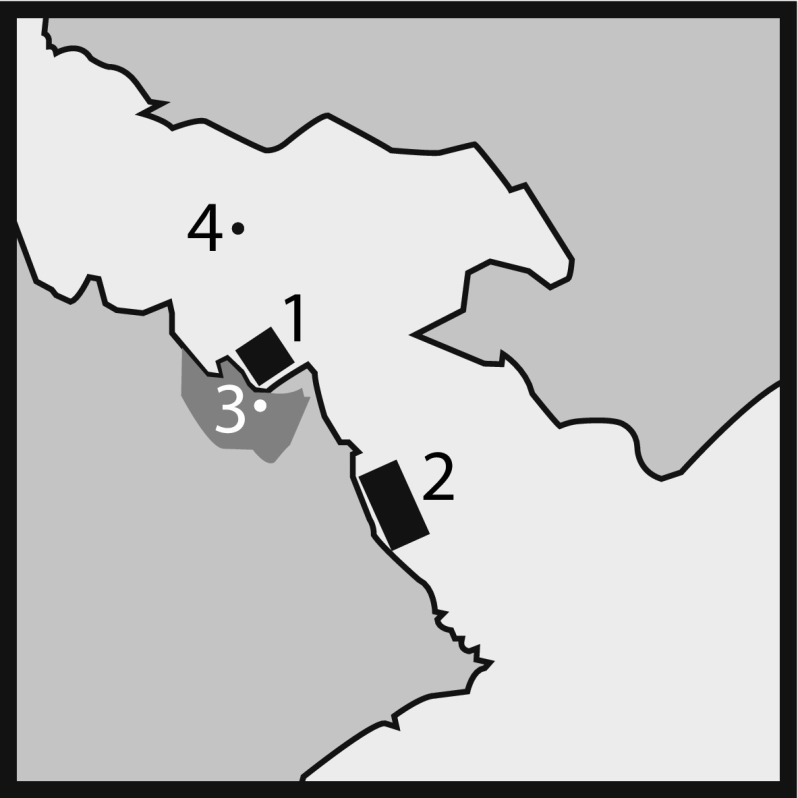
Schematic (not to scale) representation of sampling locations. (*1*) Main production area, (*2*) alternative winter harvest site, (*3*) depuration tanks and (*4*) WWTP discharge point. Approximate distance from (*1*) to (*4*) is 1.5 km and (*2*) to (*4*) is 4.5 km

During the production, Pacific oysters (*Magallana gigas*) were generally harvested from Site 1 during the months of April to September inclusive. Over the winter months (October to March, inclusive), production switched to Site 2, an alternative category A site situated at the estuary mouth and approximately 4.5 km away from the WWTP discharge outlet (Fig. 1). In September 2014 and again in September 2015, large consignments of oysters were transferred from Site 1 to Site 2 prior to any significant norovirus contamination occurring in Site (1). On occasion, throughout both winter periods, a number of further consignments of oysters were transferred to Site (2) Oysters moved during the winter months were relocated in Site 2 for at least 2 months before harvesting and were therefore considered to have equilibrated to the norovirus concentrations associated with Site 2 before harvest took place.

Following harvest from sites 1 (summer), and 2 (winter), all oysters were depurated before placing on the market. During the summer, oysters were depurated for 2–3 days at ambient temperatures. During the winter months, oysters were depurated for between 2 and 9 days at elevated temperatures.

### Depuration Procedures

Depuration undertaken by the shellfish producer was routinely performed in recirculating tanks of seawater using ultraviolet (UV) disinfection. In total, seven depuration tanks were used during the study period. Tank dimensions were approximately 6 by 0.7 by 1.3 m. The maximum volume of each tank was 6000 L of seawater pumped from a nearby pit on the estuary shore that flooded during high water. This seawater was filtered and UV treated prior to entering the depuration system. The seawater flow rate was approximately 10 m^3^/h providing effective aeration and adequate dissolved oxygen levels in the tanks to allow active functioning of the oysters. Each tank was fitted with 4 UV sterilisation lamps (55 W, UVc 200–280 nm) and a set of three 300 W aquarium water heaters, which were used when the seawater temperature dropped below 12 °C. During the study, the average seawater temperature measured during depuration in winter (October to March) was 13.3 °C (min = 8.1 °C and max = 19.7 °C). Prior to depuration, oysters were washed briefly and placed in shallow, open mesh plastic trays (8–10 kg of oysters per tray). Trays were stacked up to four layers high, with a maximum of 800 kg of oysters placed in each of the tanks for between 2 and 9 days.

### Oyster Sampling

Between January 2015 and April 2016, samples consisting of 10 live oysters were collected at the three production points on a weekly basis. Sampling schedules varied throughout the study period depending on season and are outlined in Table 1.

**Table 1:** The study sampling schedule

	Winter 2014/2015	Summer 2015	Winter 2015/2016
	January to March 2015	April to October 2015	November 2015 to March 2016
Site 1	1 (13)	2 (50)	2 (42)
Site 2	2 (26)	1 (36)	2 (39)
Depurations (total) of which	5 (62)	2 (89)	4 (62)
Short term (2–3 days)	3 (36)	2 (89)	2 (38)
Long term (7–9 days)	2 (26)	–	2 (24)

Oysters were sampled weekly from the three designated sampling points: Site 1, Site 2 and following depuration (short term and long term)

The number of samples collected weekly from each site is indicated. The total number of samples collected from each site is shown in brackets. All samples consisted of 10 live oysters

All oyster samples collected during the study period were transported to the laboratory under chilled conditions (< 15 °C) and received within 48 h of harvesting. Analysis began within 24 h of receipt to the laboratory.

#### Winter Sampling Schedule

Between January and March 2015, a sample was collected from the unused Site 1 and duplicate samples were collected from Site 2 each week. In total, five samples were collected weekly from the depuration tanks during the winter months. This consisted of triplicate samples of oysters purified for 2–3 days (short-term depuration) and duplicate samples of oysters depurated for 7–9 days (long-term depuration).

Between November 2015 and March 2016, duplicate samples were collected weekly from Site 1 and 2 as well as following short-term and long-term depuration.

In total, 55 samples were taken from Site 1 and 65 samples from Site 2 during the winter months. In the same period, 74 and 50 samples were collected following short-term (2–3 days; mean 2.42 days) and long-term (7–9 days; mean 7.24 days) depuration, respectively.

#### Summer Sampling Schedule

Sampling frequency was reduced during the summer months in line with the expected reduction in norovirus detection rates. Between April and October 2015, a single sample was collected weekly from the unused Site 2, duplicate samples were harvested from Site 1 and following short-term depuration.

In total, 50 samples were collected from Site 1 and 36 samples from Site 2 during the summer months. In the same period, 89 samples were collected following short-term depuration.

### Preparation of Oyster Samples for Norovirus Analysis

Oysters were prepared in accordance with ISO 15216-1:2017 ( 2017).

Briefly, oysters were cleaned under running potable water. Ten oysters per sample were shucked and the digestive tissues (DT) dissected out. The dissected DT was finely chopped using a sterile razor blade and mixed well. Two grams of DT was then spiked with 10 µl of the internal process control (IPC) virus (Mengo virus strain MC_0_) for evaluation of virus extraction efficiency similar to that described by Costafreda et al. (2006) and treated with 2 ml Proteinase K (100 µg ml^−1^). Samples were incubated at 37 °C for 60 min with shaking at 150 rpm followed by 15 min at 60 °C. Finally, after centrifugation at 3000×*g* for 5 min, supernatants were retained for RNA extraction.

### Viral RNA Extraction

RNA was extracted from 500 µl of the DT supernatants using NucliSENS® magnetic extraction reagents (bioMérieux) and the NucliSENS® MiniMAG® extraction platform and eluted into 100 µl of elution buffer. RNA extracts were stored at − 80 °C until the RT-qPCR analysis was conducted. RNA was also extracted from 10 µl of the IPC sample for evaluation of extraction efficiency. A single negative extraction control (water only) was processed alongside the oyster samples.

### Determination of the Norovirus Concentration Using One-Step qRT-PCR

Oysters were analysed for the norovirus concentrations using standardised quantitative real-time reverse transcription PCR (RT-qPCR) (“ISO 15216-1:2017” 2017).

RT-qPCR analysis was carried out using the Applied Biosystems AB7500 instrument (Applied Biosystems, Foster City, CA) and the RNA Ultrasense one-step qRT-PCR system (Invitrogen). The reaction was prepared by combining 5 µl of the extracted RNA sample and 20 µl of the reaction mix containing 500 nM forward primer, 900 nM reverse primer, 250 nM sequence specific probe, 1x ROX reference dye and 1.25 µl of enzyme mix. Previously described primers QNIF4 (da Silva et al. 2007), NV1LCR (Svraka et al. 2007) and TM9 probe (Hoehne and Schreier 2006) were used for the detection of norovirus GI, and QNIF2 (Loisy et al. 2005), COG2R (Kageyama et al. 2003) and QNIFS probe (Loisy et al. 2005) were used for the detection of norovirus GII. The Mengo110, Mengo209 primers and Mengo147 probe were used in IPC assay (Pinto et al. 1999). The 96-well optical reaction plate was incubated at 55 °C for 60 min, 95 °C for 5 min, and then 45 cycles of PCR were performed, with 1 cycle consisting of 95 °C for 15 s, 60 °C for 1 min and 65 °C for 1 min. All samples were analysed for norovirus GI and GII in duplicate. All control materials used in the RT-qPCR assays were prepared as described by Flannery et al. (2012). To enable quantification of norovirus RNA in copies per µl, a log dilution series of the norovirus GI and GII DNA plasmids (ranging from 1 × 10° to 1 × 10^5^ copies/µl) were included in duplicate on each RT-qPCR run. The number of RNA copies in norovirus-positive samples was determined by comparing the *C*_*T*_ value to the standard curves. The final concentration was then adjusted to reflect the volume of sample analysed and expressed as the number of detectable virus genome copies per gram of DT.

The presence of inhibitors was checked by spiking an additional 5 µl of each sample RNA with 1 µl of either norovirus GI or norovirus GII external control RNA (ECRNA; 10^5^ RNA transcripts/µl). The threshold cycle (*C*_*T*_) value obtained for samples spiked with the ECRNA was compared to the results obtained in the absence of the sample (5 µl of water used instead) and used to estimate RT-PCR inhibition expressed as a percentage. In accordance with ISO 15216-1:2017, oyster samples with RT-PCR inhibition below the 75% were accepted for inclusion in this study.

Extraction efficiency was assessed by comparing the *C*_*T*_ value of the sample spiked with IPC virus to a standard curve obtained by preparing log dilutions of the RNA extracted from 10 µl Mengo virus, and was subsequently expressed as percentage extraction efficiency. Samples with the extraction efficiency greater than 1% were accepted for inclusion in this study (“ISO 15216-1:2017” 2017).

No template controls (water only) and negative extraction controls (blank sample carried through the RNA extraction step) were included in each RT-PCR analysis in order to control for cross-contamination.

### Data Analysis

The limit of quantification (LOQ) for both, norovirus GI and norovirus GII assays was 100 genome copies/g. Results were presented as total norovirus concentration calculated as a sum of GI and GII results. To facilitate the statistical analysis of the results, any result demonstrating a norovirus concentration < LOQ was assigned a value of half of the LOQ value (50 genome copies/g) for each genogroup.

An unpaired, two-tailed *t* test was performed in Microsoft Excel to compare the norovirus concentration results obtained from the three sampling points. Results with values *p* < 0.05 were deemed statistically significant.

## Results

**Fig. 2:**
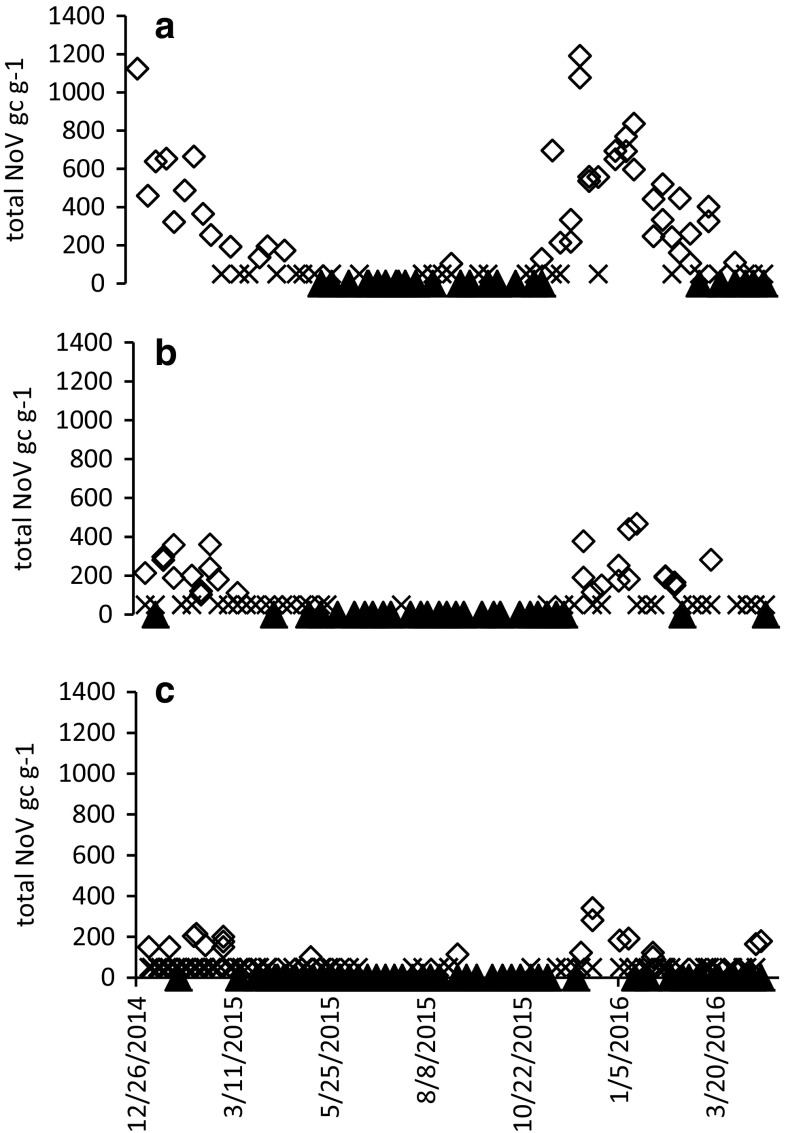
Total norovirus (genogroup I and II) concentrations in genome copies/g detected in oysters from **a** Site 1—main production area, **b** Site 2—alternative winter harvest site and **c** post depuration (2–9 days). Samples were collected weekly from the three production points between January 2015 and April 2016. Samples in which norovirus was not detected are represented by a triangle, samples containing < 100 genome copies/g (< LOQ) are represented by an X and diamonds indicate samples with results greater than 100 genome copies/g

Norovirus concentrations in oysters followed a clear seasonal trend at both Sites 1 and 2 (Fig. 2). Peak norovirus RNA concentrations were detected in oysters in the winter during the period between November and March, while norovirus was absent, or present at concentrations below 100 genome copies/g, in all but one oyster sample in the remainder of the year. This seasonal trend was also present in oysters following depuration. Norovirus RNA concentrations detected in oyster samples from Site 1 and 2 were similar during both the winter periods covered by this study. The peak concentrations of norovirus in oysters from Site 1 were 1124 genome copies/g in January 2015 and 1191 genome copies/g in December 2015. In oysters from Site 2, the peak concentrations of norovirus were 361 genome copies/g in February 2015 and 467 genome copies/g in January 2016.

During the winter periods, norovirus detection rate in oysters at Site 1 was at 91% compared to 89% at Site 2, and 81% in marketed oysters following depuration (Table 2).

**Table 2:** Norovirus detection rates and mean total norovirus RNA concentrations in oyster samples

	Production area		Marketed^a^ oysters	Depuration period	
	Site 1 (*n* = 55)	Site 2 (*n* = 65)	*n* = 124	Short^b^ (*n* = 74)	Long^b^ (*n* = 50)
Percent positive	91	89	81	84	78
Mean NoV conc. gc/g	353	117	58^c^	65^c^	47^c^
Percent of positive samples < LOQ	20	49	69	68	70
Min–max NoV conc. gc/g	n.d.–1191	n.d.–467	n.d.–341	n.d.–341	n.d.–204

Total norovirus RNA concentrations in each sample, calculated as a sum of GI and GII genogroup results were used to determine the mean concentration. Samples were collected from Site 1, Site 2 and post-depuration during winter periods of January to March 2015 and October 2015 to March 2016

*n.d* not detected

^a^Oysters following depuration for either short- or long-term periods

^b^Short and long depuration periods were 2–3 days (mean 2.4 days) and 7–9 days (mean 7.2 days), respectively

^c^Mean norovirus concentration in genome copies/g determined by assigning a value of 50 to all < LOQ results

However, there was a significant gradation in the norovirus RNA concentrations detected in oysters sampled at Site 1 and 2, and following depuration during the same period (*p* < 0.05). The maximum and mean norovirus RNA concentrations detected in oysters from Site 1 were 1191 and 353 genome copies/g, respectively. This is compared with maximum of 467 and mean of 117 genome copies/g at Site 2. Following depuration, these figures were reduced to 341 and 58 gc/g (< LOQ), respectively.

**Fig. 3:**
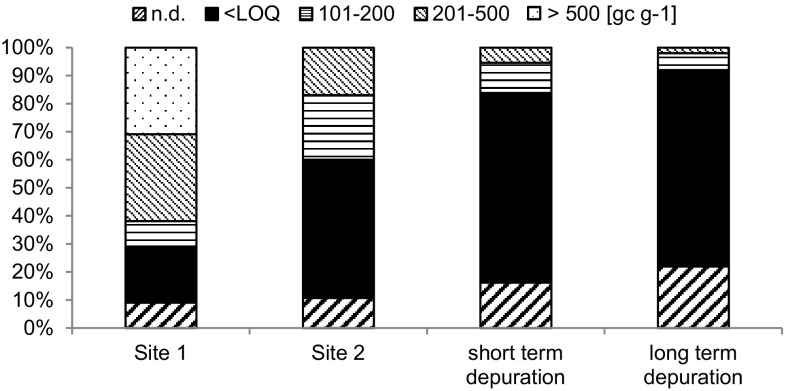
Impact of winter management interventions on norovirus concentrations in oysters. Total norovirus concentrations detected in oysters at Site 1 (*n* = 55), Site 2 (*n* = 62) and following short-term (*n* = 74) and long-term depuration (*n* = 50) were assigned to arbitrary concentration intervals; bottom to top: n.d.; <LOQ; 101–200; 201–500 and > 500 genome copies/g. The percentage numbers of each sample falling within an assigned category are given for each site. *n.d*. not detected

To determine the theoretical compliance of oyster batches with potential future acceptable limits for norovirus in oysters, the percentage of oyster samples conforming to arbitrarily selected norovirus concentration intervals was determined for each point in the production process (Fig. 3). Norovirus RNA concentrations in excess of 500 genome copies/g were detected in 31% of oyster samples taken at Site 1 during the winter, whereas no oysters from Site 2 or following depuration contained norovirus RNA concentrations above that value. In the same period, a total of 60% of oyster samples contained norovirus RNA concentrations below 100 genome copies/g at Site 2 compared with 29% of samples from Site 1. Following both short- and long-term depuration, the frequency of samples containing less than 100 norovirus genome copies/g increased to 88% overall. The percentage of samples containing norovirus concentration below 100 genome copies/g in oysters subjected to depuration for 2–3 days was 84% compared with 92% of oysters depurated for 7–9 days.

## Discussion

During this study, norovirus detection in oysters followed a clear seasonal trend with both a higher frequency of detection and increased norovirus RNA concentrations detected in the winter season (November to March). This confirms finding in other studies conducted in Europe (EFSA 2012; Flannery et al. 2009; Lowther et al. 2012b) and highlights a need for additional targeted risk management interventions at this time of year. It is significant that high concentrations (> 1000 genome copies/g) were detected in oysters sampled from the main harvest area (Site 1) during the high-risk winter months. Similar norovirus RNA concentrations have previously been detected in oysters associated with illness outbreaks (Doré et al. 2010; Lowther et al. 2012a; Rajko-Nenow et al. 2013). Based on *E. coli* monitoring data and despite the high concentrations of norovirus detected, Site 1 was classified as ‘Class A’ harvesting area under EU regulations. Shellfish harvested from a Class A area can be placed directly on the market even during the high-risk winter period. In 2010, oysters produced at Site 1 were associated with a major illness outbreak (Doré et al. 2010). At that time, oysters were marketed directly from Site 1 without additional post-harvest treatment or following minimal depuration periods (< 48 h) and without temperature control. Given the norovirus RNA concentrations detected in this study, it is likely that if oysters from Site 1 had continued to be placed on the market directly, with or without minimal treatment, further illness outbreaks would have occurred.

Following the outbreak associated with oysters harvested from Site 1, the producer introduced additional management controls which included the use of an alternative harvest site (Site 2) during the winter (November to March) and depuration for extended periods under controlled temperature conditions. Nevertheless, the additional controls resulted in a limited reduction in the frequency of norovirus detection in marketed oysters during winter with norovirus detected in 91, 89 and 81% of oysters from the main production site (Site 1), alternative harvest site (Site 2) and following depuration, respectively. However, a reduction in norovirus concentration was observed in the oysters following implementation of the management procedures. It was notable that the majority (84%) of oyster samples following depuration contained norovirus RNA concentrations below the LOQ. Therefore, while management procedures employed during this study did not significantly lower the frequency of norovirus detection in marketed oysters, they reduced the concentration of norovirus copies, to which consumers were exposed.

The overall impact on potential illness cases of implementing the management procedures employed during this study cannot be determined and remains uncertain. Norovirus has a low infective dose with one study based on a human exposure trial reporting the 50% human infectious dose (HID_50_) to be between 18 and 1015 genome equivalents for norovirus GI.I (Teunis et al. 2008). In addition, illness outbreaks following the consumption of oysters with norovirus RNA concentrations < 100 genome copies/g have been reported (Thebault et al. 2013). Therefore, the low concentrations of norovirus as detected in market ready oysters in this study could still have the potential to make consumers ill. However, by its nature, the RT-qPCR method detects genome copies only and does not distinguish between infectious and non-infectious virus copies. This further increases the uncertainty of the illness outcome for a given concentration of norovirus genome copies. In addition, a second study has reported a higher HID_50_ for norovirus GI.I at 1320 (95% CI 440–3760) genome equivalents for susceptible individuals (Atmar et al. 2014). It is clear that there remains significant uncertainty regarding the dose response models developed for norovirus thus far. This makes it extremely difficult to determine the likely illness outcomes associated with a given concentration of norovirus genome copies detected in oysters. In reality, the illness outcome for any given norovirus concentration in oysters will vary depending on a range of factors including differences between norovirus genotype and host susceptibility based on immunity, and genetic susceptibility (Noda et al. 2008). A very significant factor may also be the type of contamination event impacting on the oysters. For example, oysters contaminated with nearby untreated sewer overflows will likely contain a higher ratio of infectious norovirus than oysters contaminated with disinfected sewage from a distant location for any given norovirus genome copy concentration. However, this will depend on the efficacy of the type of wastewater treatment applied, for example, recent evidence suggests that chlorine-based sewage treatment may not substantially inactivate norovirus (Kingsley et al. 2017). Despite these uncertainties, a clear relationship between increasing genome copy concentrations in oysters and illness outcome has been reported (Lowther et al. 2012a). It is apparent that the risk of an illness event rises with increasing number of genome copies present, even if this increased risk is not quantifiable. Conversely, the decrease in norovirus concentration due to the procedures reported here must therefore be considered likely to reduce the risk of illness associated with oyster consumption. Further, the fact that there were no reported incidences of illness associated with consumption of more than 3 million oysters sold from this production site over the study period would appear to suggest that the health risk was indeed reduced. However, it is also the case that the vast majority of norovirus-related gastroenteritis is unreported and one large-scale study indicates that approximately only 1 in 300 cases of norovirus gastroenteritis occurring in the community may be recorded in the national statistics (Tam et al. 2012). Therefore, the lack of documented incidences during this study does not, in itself, indicate a lack of risk. It is possible that sporadic unreported illness occurred following the consumption of oysters from the site particularly during the high-risk winter period. Clearly, further characterisation of the relationship between norovirus genome copy concentrations determined in oysters by RT-qPCR and illness outcome is required to be able to fully assess the impact of management procedures adopted by producers.

In this study, harvesting was switched to a less contaminated site during the high-risk winter period. Monitoring of both the main harvest and the alternative site for norovirus confirmed that on average oysters in the alternative harvest site contained lower concentrations of norovirus. This was despite the fact that there was no difference in the number of oyster samples positive for norovirus. It is therefore worth noting that, in this context, the standardised RT-qPCR method provided a robust and reliable tool to allow characterisation of the two harvest areas in relation to the extent of norovirus contamination. Initial virus concentration has been demonstrated to have an impact on the outcome of virus depuration i.e. the higher the initial virus concentration, the higher the final virus concentration if all other parameters are equal (McLeod et al. 2017). By using the alternative site, norovirus RNA concentrations in oysters sent for depuration were reduced compared to oysters from the main harvest area. This would undoubtedly contribute to the fact that the majority of norovirus-positive oyster samples contained norovirus RNA concentrations below 100 genome copies/g following depuration.

Depuration is one of the most widely practiced post-harvest treatments during the production of raw oysters (Lees et al. 2010). The process was originally designed in the beginning of the twentieth century to prevent bacterial illness associated with shellfish consumption. It has been documented on numerous occasions that depuration is unable to achieve complete elimination of viruses (reviewed by McLeod et al. 2017). We confirm this finding here with the majority of oysters still containing norovirus following depuration during the high-risk winter period. However, depuration as practiced in this study did have an overall impact on the concentrations of norovirus in oysters. Previous studies have indicated that the time and seawater temperature are both factors that may influence virus reduction during bivalve shellfish depuration (Lees et al. 2010). Nevertheless, the minimum depuration times and temperatures are not stipulated in EU regulation. In Ireland, in common with many other countries in Europe, it is recommended that depuration should be carried out for a minimum of 42 h at temperatures not less than 8 °C. This minimal time temperature regime has been shown to effectively reduce *E. coli* but achieves a minimal reduction of viruses. During this study, extended depuration periods of up to 9 days were applied during the winter season. In addition, minimum depuration temperatures were generally significantly above the recommended minimum with the mean temperature over all depuration cycles of 13.3 °C during the winter. It is notable that these depuration conditions were routinely applied by the producer on a commercial basis without any deterioration of shellfish quality and were considered economically viable.

Interestingly, even under these enhanced conditions (elevated temperature and extended time), only a slight drop in the number of oyster samples containing norovirus was observed. However, there was a notable drop in the average concentration of norovirus in oysters before and after depuration with most (90%) of oysters following depuration for 7–9 days containing < 100 genome copies/g. This is compared with just 55% of oysters containing norovirus RNA concentrations < 100 genome copies/g prior to depuration. A possible explanation for the reduction in norovirus RNA concentrations observed in this study is that norovirus was sequestered into tissues outside of the digestive tissue. Given that the ISO standard method used in this study examines the digestive tissue only, we cannot determine from this study whether this is the case but we can find no evidence in the literature of such sequestration into alternative tissues. On average oysters purified for 7–9 days contained slightly lower concentrations of norovirus than oysters depurated for 2–3 days indicating that depuration for extended periods may further reduce norovirus RNA concentrations. However, the additional reduction in norovirus RNA concentrations in oysters depurated beyond the 3 days was not significantly different (*p* > 0.05) and its value as an added public health control is questionable. The limited value of depuration periods extended beyond the 3 days as observed here is consistent with laboratory-based studies reported elsewhere for other shellfish species (Polo et al. 2015) and oysters (McLeod et al. 2017). Interestingly, laboratory-based depuration studies have demonstrated that virus reduction is a two-phase process (Polo et al. 2014). The first phase is a relatively rapid process related directly to shellfish filtration rate. However, subsequent low-level norovirus persistence is associated with a second phase demonstrating a significantly slower reduction rate where viruses appear to be refractory to the initial depuration process, possibly because they are intrinsically bound to specific norovirus receptors in the oyster tissue.

In summary, in the absence of current regulatory standards, we believe that a site-specific management approach, such as described here and supported by norovirus monitoring, can reduce consumer exposure to norovirus genome copies. This may provide additional, if not complete, consumer protection. However, despite the anecdotal evidence presented here, i.e. lack of illness reports, it is not possible to determine the public health benefits of this approach. Therefore, there remains a clear requirement for further work to better characterise the relationship between norovirus RNA concentrations in oysters as judged by RT-qPCR and illness outcomes.
